# Stage IAE Follicular Lymphoma of the Breast: Case Report and Review of the Literature

**DOI:** 10.1155/2013/597527

**Published:** 2013-05-08

**Authors:** Raymon Patron, Edward F. Miles

**Affiliations:** ^1^Department of Internal Medicine, Naval Medical Center Portsmouth, 620 John Paul Jones Circle, Portsmouth, VA 23708, USA; ^2^Division of Radiation Oncology, Department of Radiology, Naval Medical Center Portsmouth, 620 John Paul Jones Circle, Portsmouth, VA 23708, USA

## Abstract

Primary lymphoma of the breast is a rare entity in the field of oncological medicine and represents <0.5% of all breast malignancies. A definitive diagnosis is obtained by excisional biopsy as the clinical and radiographical presentation is similar to the more common primary breast carcinoma. Unlike primary breast carcinoma, localized radiation therapy is the mainstay of treatment. We report on a case of primary follicular lymphoma of the breast in a 67-year-old Caucasian woman treated with localized radiation as well as coinciding literature review regarding outcomes of different treatment modalities.

## 1. Introduction

Follicular lymphoma is the second most common lymphoma diagnosed in the United States, comprising 20% of all non-Hodgkin lymphomas and 70% of all indolent lymphomas [1]. Median age at diagnosis is 60 years with a slight female predominance [2, 3]. Most patients present with asymptomatic lymphadenopathy, and more than 90% present with Stage III or IV disease [4]. Primary breast lymphoma is much less common and represents 1% of all non-Hodgkin lymphomas [5] and <0.5% of all breast malignancies [6]. 

Management of follicular lymphoma depends on the stage; for early stage disease (I and II), current guidelines recommend the consideration of 2,400–3,000 centiGray (cGy) involved-field radiation therapy for nodal sites and involved-organ radiation therapy for extranodal sites [7].

We report a case of successful local control of an incidental follicular lymphoma of the breast diagnosed at excisional biopsy. 

## 2. Case Report

**Figure 1:**
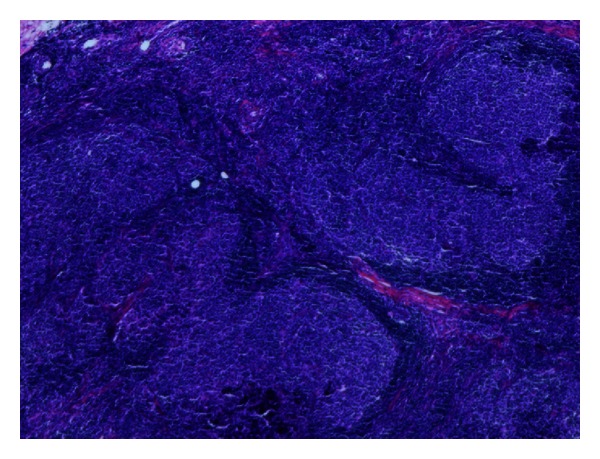
Follicular lymphoma of the breast at 4x magnification in hematoxylin and eosin stain.

**Figure 2:**
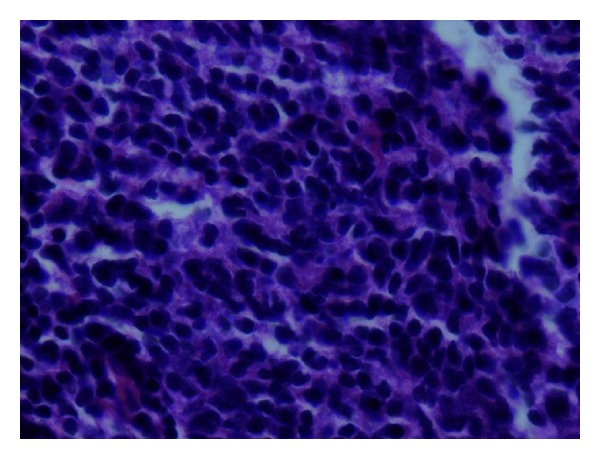
Follicular lymphoma of the breast at 40x magnification in hematoxylin and eosin stain.

The patient was a 67-year-old Caucasian female who self-palpated a 1 cm firm nodule in the midsuperior portion of the right breast. Her screening mammogram was normal eight months before. Ultrasound evaluation demonstrated a mixed density lesion measuring 1 cm in greatest dimension, directly overlying the pectoralis muscle. She underwent excisional biopsy which demonstrated Grade 2 follicular lymphoma (Figures 1 and 2). Due to the unusual diagnosis, her case was reviewed at a multidisciplinary tumor board and discussed in detail with radiology, pathology, general surgery, radiation oncology, and medical oncology specialists.

She subsequently underwent a complete workup per the national comprehensive cancer network (NCCN) guidelines, [8] including a bone marrow biopsy which was normal and a positron emission tomography/computed tomography (PET/CT) study which demonstrated nither regional or distant adenopathy nor residual PET-avid disease in the right breast. She denied fevers, night sweats, or significant weight loss and was staged with Stage IAE follicular lymphoma of the right breast. Radiation therapy was offered to limit the risk of local recurrence with close monitoring for systemic recurrence.

**Figure 3:**
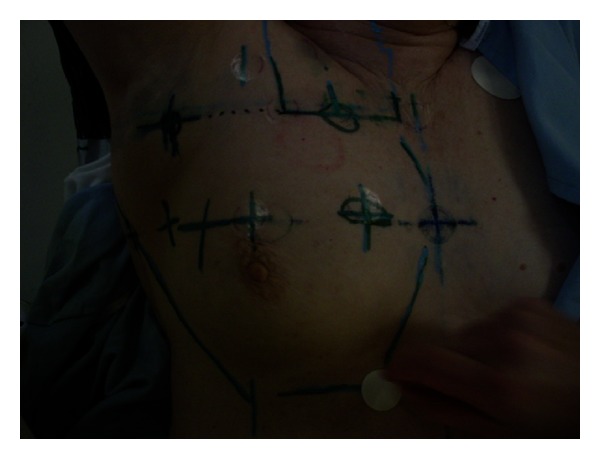
Anterior light field markings for right breast tangents with separate supraclavicular field.

**Figure 4:**
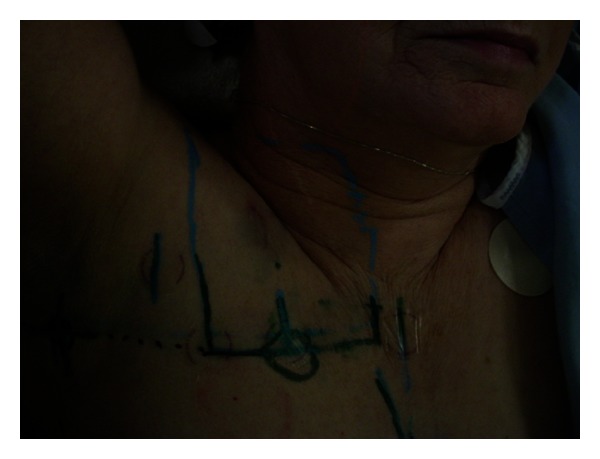
Supraclavicular light field with central scar from tumor resection.

Standard breast tangents were used with the medial border at the sternal notch, the lateral border at the midaxillary line, the inferior border two centimeters below ipsilateral breast tissue, and the superior border placed at the inferior border of the clavicular head. Due to the location of the tumor bed in this position (Figure 3), a separate supraclavicular field, matched to the nondivergent superior border of the tangents, was required to ensure the coverage of this area, which included the tumor bed (Figure 4). A separate axillary field was not used, but the tangent fields were noted to cover levels one and a portion of two in the ipsilateral axilla. The tumor bed and adjacent critical structures including the lungs and heart were contoured on a slice-by-slice basis. 

A field-in-field technique with dual energy photons (6 and 10 MV) delivered 200 cGy fractions per day to the tangent fields, while a 6 MV photon beam was used to treat the supraclavicular field. Both fields received a total dose of 3,000 cGy over 19 days. She completed the treatment as planned and experienced only Grade 1 radiation dermatitis. 

A PET/CT scan performed four years after completion of her lymphoma therapy demonstrated no evidence of recurrent lymphoma.

## 3. Discussion

Primary breast lymphoma (PBL) is a rare disease process similar clinically and radiologically to common primary breast carcinoma [9]. The patient must meet certain diagnostic criteria to make the diagnosis of PBL including lymphomatous infiltrate of breast tissue with no other organ or nodal involvement at the time of diagnosis with the exception of ipsilateral axillary lymph nodes [10]. Primary follicular lymphoma of the breast as described in this case is the second or third most prevalent primary breast lymphoma preceded by diffuse large cell lymphoma (DLCL) and marginal zone lymphoma (MZL) [11–13]. We reviewed three studies examining the outcome of treatment modalities regarding this specific primary breast malignancy. 

Martinelli et al. report on a multicentered study of 36 cases of follicular primary breast lymphoma over 23 years. Patients were resected with surgery, chemotherapy, and radiation therapy alone or in combination. Data collected showed 14 patients out of the population relapsed after a median interval of 26 months. Eight patients with local disease treated with radiation as a first-line treatment showed no recurrence in irradiated fields. Median followup at approximately 44 months showed 8/36 died from the disease with cause-specific survival for follicular primary breast lymphoma: 84% at 3 years, 79% at 5 years, and 66% at 10 years [11].

Ganjoo et al. report on a case study involving 37 cases of breast lymphoma including DLBCL, MZL, and SLL. Seven out of 37 demonstrated follicular lymphoma by biopsy. Of the 7, one case of follicular lymphoma staged IE, similar to the case presented, was treated with localized radiation therapy alone, receiving 3600–5040 cGy including the breast and axilla. The remaining 6 cases of follicular lymphoma with IIIE, IV were treated with a combination of radiation and CHOP/CVP or Rituximab. The patient treated for localized disease remained NED after a median followup of 5.8 years. The 5-year overall survival rate for patients with indolent lymphomas treated with CHOP/CVP or Rituximab was found to be 92% [12].

Talwalkar et al. described 106 cases of lymphomas involving the breast. Of the 106, 15 were documented as disseminated follicular lymphoma. Of the forty-five patients, received chemotherapy 38 alone, 4 with adjuvant radiation therapy and 3 with combination chemotherapy, lumpectomy and radiation therapy. The 15 documented cases of follicular lymphoma, showed a median overall survival of only 24 months. However, the cases described in this study were those of disseminated disease involving the breast not necessarily the primary breast lymphoma described in this case [13].

In conclusion, most patients with follicular lymphoma of the breast who present with early stage local disease responded very well to definitive radiation therapy [11, 12]. This patient's tumor bed was in the superior breast and required a separate supraclavicular field to treat the entire involved organ and tumor bed. There is no evidence of disease 4 years after completion of definitive therapy.
